# Children’s HeartLink Certifies Center of Excellence in Brazil

**DOI:** 10.21470/1678-9741-2023-0485

**Published:** 2024-05-10

**Authors:** Ulisses Alexandre Croti, Bruna Cury Borim, Andreas Tsakistos, Jackie Boucher, Bistra Zheleva

**Affiliations:** 1 CardioPedBrasil® - Centro do Coração da Criança at Hospital da Criança e Maternidade São José do Rio Preto, São Paulo, Brazil (FUNFARME/FAMERP); 2 Children’s HeartLink, Minneapolis, Minnesota, United States of America

**Fig. 1:**
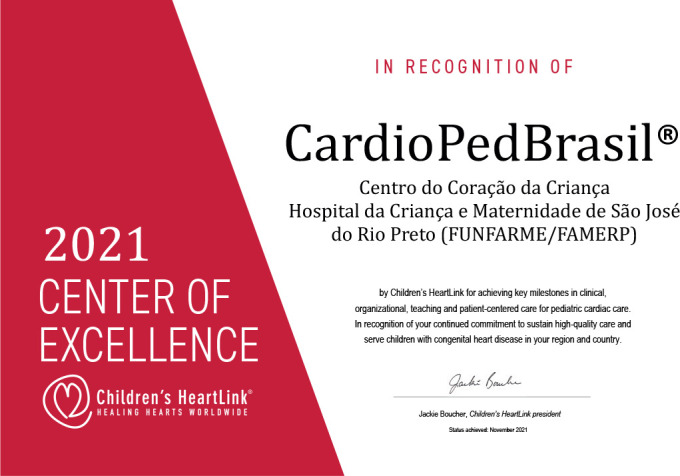
Recognition of CardioPedBrasil® as a Children’s HeartLink Center of Excellence presented in person by President Jackie Boucher at the 6^th^ Children’s HeartLink International Meeting in Brazil in September 23^rd^, 2023.

During the 6^th^ Children’s HeartLink International Meeting in Brazil, held on September 23, 2023, focusing on Quality and Education in Pediatric Cardiology and Cardiovascular Surgery, the CardioPedBrasil® - Centro do Coração da Criança of the Hospital da Criança e Maternidade (HCM) of São José do Rio Preto/SP (FUNFARME/FAMERP) was recognized as the Children’s HeartLink’s first Center of Excellence in Brazil and in Latin America (Figure 1). Children’s HeartLink is a US-based nongovernmental organization (NGO) dedicated to building capacity and advocating for better access to pediatric cardiac care (https://childrensheartlink.org/)^[1]^.

The event streamed live to 18 Children’s HeartLink partner sites a surgical correction of a complete atrioventricular septal defect performed by surgeon Dr. David Overman. It gathered over 300 professionals and cardiovascular surgeons from all over Brazil, including former presidents of the Brazilian Society of Cardiovascular Surgery, Dr. José Wanderley Neto, Dr. José Teles de Mendonça, and Dr. Fernando Antonio Lucchese, as well as the current president-elect, Dr. Vinicius Nina.

Children’s HeartLink named the CardioPedBrasil® - HCM a Children’s HeartLink Center of Excellence after the hospital achieved a series of continuous improvements since the beginning of the partnership in 2009^[1]^. Medical volunteers from Mayo Clinic, Rochester, Children’s Hospitals and Clinics Minnesota, Boston Children’s Hospital, and Seattle Children’s Hospital, among others, playing an active training and mentoring role, contributed to this progress^[2]^.

CardioPedBrasil® is strategically located within a pediatric hospital with maternity services, a state and national referral hospital for congenital heart disease, facilitating high quality care of pregnant women and newborns with complex congenital heart diseases. The location also allows patient care to be provided by all the necessary specialized professionals and diagnostic exams such as magnetic resonance imaging, computerized tomography scans, a hemodynamics laboratory led by a pediatric cardiologist, and fetal and pediatric echocardiography available 24 hours.

With a 30-bed Pediatric Cardiac Intensive Care Unit, 21- bed Pediatric Cardiac Ward, and two exclusive and fully equipped surgery rooms with state-of-the-art technology, ensuring safety and precision in pediatric cardiovascular procedures, CardioPedBrasil® is able to accommodate over 450 surgical patients annually from across the country. Patients and their families receive follow-up care at the pediatric cardiology outpatient clinic and, if needed, have access to free accommodation and meals in one of our two nearby housing facilities, Associação dos Amigos da Criança com Câncer ou Cardiopatia (https://amicc.com.br/) and Casa Thales (https://casathales.com.br/).

CardioPedBrasil® also created an innovative educational website (www.cardiopedbrasil.com.br) that offers entirely free and high-quality information, sharing knowledge acquired over more than 20 years of experience and over 6,000 pediatric operations. More than 300 video lectures, downloadable documents and images, and content across various social media platforms are available, aiming to empower healthcare professionals and caregivers of children with congenital heart disease across Brazil.

To be named a Children’s HeartLink Center of Excellence requires meeting several goals set by the NGO. Throughout the years of collaboration, there has been a noticeable transformation in the interdisciplinary team’s culture, especially in communication, teamwork, and nursing empowerment. This effort resulted in the review and improvement of all processes and protocols, aiming to enhance morbidity and mortality rates^[3,4]^.

Significant reduction in infection and mortality rates, establishment of a daily academic program for the interdisciplinary team, and formal professional training programs such as pediatric cardiovascular residency, fellowships in pediatric cardiac intensive care unit, pediatric cardiovascular surgery, fetal, transesophageal, and pediatric echocardiography, as well as perfusion and pediatric perfusion training, were among the accomplishments^[4]^.

Part of the Children’s HeartLink and the medical volunteers’ efforts focused on convincing the hospital administration to make significant investments in human resources to meet the requirements for adequate staffing. Currently, the interdisciplinary team includes over 200 continuously trained professionals, exclusively dedicated to caring for hospitalized patients with congenital heart disease.

The Institution prioritized ongoing education of the interdisciplinary team, supported the development of a specialized in-hospital pediatric cardiac nursing orientation developed by a pediatric cardiology nurse educator, and hired 68% bachelor’s degree nursing professionals. Also, 23% of their registered nurses have graduated from the first and only postgraduate course for pediatric cardiology and cardiovascular surgery nursing in Brazil, created and developed by CardioPedBrasil®, now in its third year and over 90 graduated nurses from around the country.

The team is regularly invited as faculty to national and international conferences and participates in collaborative research publications serving as a thought leader in the field of pediatric cardiac care in low-resource settings. Recently, they contributed to the development and publication of global recommendations for developing pediatric and congenital heart disease services in low-income and middle-income countries^[5]^.

**Fig. 2:**
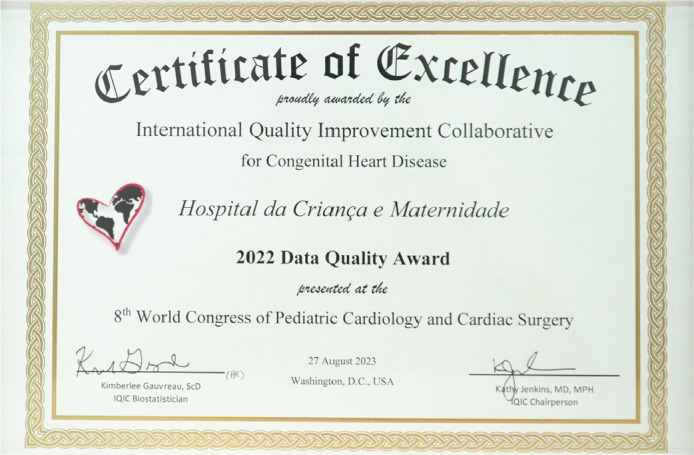
International Quality Improvement Collaborative for Congenital Heart Disease (IQIC) Quality Excellence Award for data collection presented to CardioPedBrasil® in Washington DC, United States of America, during the IQIC Session at the 8^th^ World Congress of Pediatric Cardiology and Cardiac Surgery in August 27^th^, 2023.

The partnership with Children’s HeartLink also supported participation in the International Quality Improvement Collaborative for Congenital Heart Disease (IQIC) registry, where our uninterrupted data submission since 2010 is regularly audited by a team from Boston Children’s Hospital at Harvard Medical School (https://iqic.chboston.org/). In 2023, CardioPedBrasil® received the quality excellence award for data collection with more than 3,500 patients included (Figure 2). Annually, the IQIC team sends a benchmarking report comparing our performance individually and with other participating sites worldwide, strengthening our understanding of areas for improvement and enabling continuous adjustments to enhance care.

With all these accomplishments, we remain committed, seeking improvements and addressing challenges in the field, aware that this certification fills us with pride and carries significant responsibility, solidifying our national role in caring for children with congenital heart disease in Brazil.
